# Quality of DNA extracted from saliva samples collected with the Oragene™ DNA self-collection kit

**DOI:** 10.1186/1471-2288-12-65

**Published:** 2012-05-04

**Authors:** Ana P Nunes, Isabel O Oliveira, Betânia R Santos, Cristini Millech, Liziane P Silva, David A González, Pedro C Hallal, Ana M B Menezes, Cora L Araújo, Fernando C Barros

**Affiliations:** 1 Histology Laboratory, Morphology Department, Institute of Biology, Universidade Federal de Pelotas (UFPel), Pelotas, Brazil; 2 Physiology Department, Institute of Biology, Universidade Federal de Pelotas (UFPel), Pelotas, Brazil; 3 Academic Doctoral Biological Sciences, Physiology Department, Institute of Health Basic Sciences, Universidade Federal do Rio Grande do Sul (UFRGS), Porto Alegre, Brazil; 4 BS in Biological Sciences (UFPel), Pelotas, Brazil; 5 Academic of Postgraduate in Biotechnology (UFPel), Pelotas, Brazil; 6 Nutrition Department, Universidade Federal de Santa Catarina, Florianópolis, Santa Catarina, Brazil; 7 Postgraduate in Epidemiology (UFPel), Pelotas, Brazil

## Abstract

**Background:**

Large epidemiological studies in DNA biobanks have increasingly used less invasive methods for obtaining DNA samples, such as saliva collection. Although lower amounts of DNA are obtained as compared with blood collection, this method has been widely used because of its more simple logistics and increased response rate. The present study aimed to verify whether a storage time of 8 months decreases the quality of DNA from collected samples.

**Methods:**

Saliva samples were collected with an OrageneTM DNA Self-Collection Kit from 4,110 subjects aged 14–15 years. The samples were processed in two aliquots with an 8-month interval between them. Quantitative and qualitative evaluations were carried out in 20% of the samples by spectrophotometry and genotyping. Descriptive analyses and paired t-tests were performed.

**Results:**

The mean volume of saliva collected was 2.2 mL per subject, yielding on average 184.8 μg DNA per kit. Most samples showed a Ratio of OD differences (RAT) between 1.6 and 1.8 in the qualitative evaluation. The evaluation of DNA quality by TaqMan®, High Resolution Melting (HRM), and restriction fragment length polymorphism-PCR (RFLP-PCR) showed a rate of success of up to 98% of the samples. The sample store time did not reduce either the quantity or quality of DNA extracted with the Oragene kit.

**Conclusion:**

The study results showed that a storage period of 8 months at room temperature did not reduce the quality of the DNA obtained. In addition, the use of the Oragene kit during fieldwork in large population-based studies allows for DNA of high quantity and high quality.

## Background

Epidemiological studies for the development of DNA biobanks have increasingly used less invasive methods for extracting genetic material, such as collection of buccal epithelial cells from saliva [1,2]. Other methods used in population-based studies to obtain DNA are collection of peripheral blood, swab and mouthwash for buccal cell collection, and FTA cards (Fluorescence Treponema Absorption) [1,3].

Successful genetic epidemiological studies depend on the extraction of DNA of adequate quantity and quality, both of which are influenced by the method and tissue used for biological material collection [4]. Koni et al. [1] recently made clear the limitations of low-quality DNA in biobanks, in addition to summarizing previous studies that used saliva collection to obtain DNA. The concentration of DNA extracted from blood leukocytes processed by saline extraction is 28.4 μg (11.3-59.5 μg) from 2 mL of blood [5], whereas the quantity of DNA obtained by saliva collection is 34.91 μg (2.20 – 122.04 μg) from 3 mL of saliva [6]. Although saliva collection provides smaller amounts of DNA than blood, this biological sampling method has been widely used, especially because it requires more simple logistics, including self-collection by study subjects and sample mailing [7-11]. Notably, saliva collection in children may provide lower amounts of DNA compared with adults [12,13]. However, DNA obtained from buccal cells by saliva or using a sponge does not interfere with the analysis of single-nucleotide polymorphisms (SNPs) [1,14,15], and it is even comparable to the material obtained by blood collection [5]. The collection of saliva from oral rinse or spit provides DNA with better quality than from buccal swabs or brush techniques [16].

Among the commercial kits used to obtain DNA from saliva, the Oragene™ DNA Self- Collection Kit assures no sample degradation, even when stored at room temperature for up to 30 months [17-19], and an average yield 110 μg of DNA from 2 mL of Oragene DNA/saliva samples [20].

Although widely used as a source of genetic material, there are some limitations for determining the amount of DNA obtained from buccal cells, because the concentration can differ between individuals and may contain non-human DNA, degraded or with contaminants. However, compared with other non-invasive methods, DNA extracted from saliva cells has proven to have the highest quality [5,16]. For DNA quantification, the fastest and least expensive method is ultraviolet (UV) spectrophotometry. Other methods for this measurement include agarose gel electrophoresis, fluorescent dyes, such as Hoechst and PicoGreens™, real- time polymerase chain reaction (RT-PCR), and hybridization techniques. Although these methods have high correlations with quantification measurements [11], they all can lead to biased quantification, especially in samples with low DNA concentration [21].

The most widely used technique for evaluating DNA quality is ultraviolet (UV) spectrophotometry with calculation of Ratio of OD differences (RAT), with an acceptable range between 1.6 and 1.8. Comparative studies of the quality of DNA obtained by blood and saliva collection verified that both methods provided results within the acceptable and recommended RAT range [5].

The present study aimed to verify whether a storage time of 8 months decreases the quality of DNA, based on the analysis of a subsample of 20% accessed by the quality control of DNA from teenagers of the 1993 Pelotas (Brazil) birth cohort study.

## Methods

Between January and August 2008, saliva samples were collected from 4,110 adolescents in the 1993 Birth Cohort in the city of Pelotas, southern Brazil [22,23], using the Oragene DNA Sample Collection Kit (OG-250 Disc Format, DNA Self-Collection Kit, Genotek, Ottawa, Ontario, Canada). The manufacturer’s stated specifications for this kit include the possibility of storing the saliva samples at room temperature for up to 5 years [19], the provision of high quality DNA, median yield of DNA of 110 μg from 4 mL of Oragene·DNA/saliva solution. The manufacturer concedes that the yield may range from 15 μg to more than 300 μg, and recommends that final concentration of DNA should be less than 200 μg/mL (200 ng/μL) [24].

The adolescents and their parents/guardians were informed of the study’s purposes and were then asked to sign an informed consent form. The present study was approved by the Research Ethics Committee at Universidade Federal de Pelotas Medical School.

The adolescents were asked to fast for at least 30 minutes before saliva collection. At their arrival at the collection site, they were asked if they were fasting. If yes, a mouthwash with water was performed and they had to wait at least 15 minutes before the sample collection. For saliva collection, the subjects were asked to rub their tongue against the inside of the mouth for 15 seconds and provide an amount of saliva up to the mark of a collection vial. After this, the vial was sealed, identified, and gently inverted for 10 times to mix saliva samples and the Oragene solution. The samples containing Oragene·DNA/saliva mixed were then taken to a laboratory where their transparency was examined, after which they were stored at room temperature until processing (after 3 days or 8 months later).

DNA was extracted within 3 days of saliva collection and again eight months later. The first extraction was performed using a standard volume of 2.0 mL Oragene·DNA/saliva mixed samples and the second one using the remaining volume of mixer in the collection vial. Both extractions were performed according to the manufacturer’s protocol. Briefly, the collected material underwent lysis with a purifying buffer provided in the kit for protein precipitation, followed by an ice bath, and DNA precipitation with 100% ethanol. The DNA was rehydrated in 300 μL of TE (Tris–HCl 1 M pH 8.0 and 0.5 M EDTA pH 8.0) for the first extraction and its equivalent for the second extraction, based on the volume of Oragene DNA/saliva solution remaining in the vial. After extraction, DNA samples was stored at 4°C for 7 days before spectrophotometric analysis and then stored in a freezer at −20°C. DNA quantity and quality was evaluated by Quality Control in 822 samples (20% of all) by ultraviolet (UV) spectrophotometry using an Eppendorf biophotometer (Eppendorf, Hamburg) at a dilution of 5:95 (10μL of sample in 190μL of Milli-Q); readings at 260 nm, 280 nm and 320 nm were performed. After the readings, the concentration was adjusted by the formula [(A260-A320) ×20×50]. The ratio between absorbance readings was calculated using the following formula, as suggested by the kit: RAT = (A260 – A320)/(A280 – A320). The yield of extraction was calculated by multiplying the concentration by the total volume of DNA solution after extraction and divided by the volume of Oragene·DNA/saliva solution processed in each extraction. The DNA quality was also assessed by the amplification rate of three fragments of different sizes, using the second sample processed individually, where two fragments were from the *IL4* gene: SNPs -rs2243250 (51 bp) and twelve CpG sites on the promoter (284 bp fragment), and analyzed by TaqMan and HRM assay, respectively. The third fragment, from adiponectin gene (518 bp) was analyzed using PCR-RFLP (data not published yet).

Descriptive and paired *t*-test analyses were performed using STATA 10.0 to establish whether there were changes in DNA yield during the sample storage period. The paired-t test was used to evaluate the effects of storage time of 8 months on DNA quality and yield of the same saliva sample (sample unit) at different times.

## Results

The description of the age and sex of individuals belonging to the study, and of the subsample, are presented in Table 1. No differences were found between the subsample and the whole cohort according to age or sex. Data of both measures DNA quantity and quality were normally distributed.

**Table 1:** Descriptive characteristics of whole cohort of subjects born in 1993, city of Pelotas, southern Brazil, and the subsample assessed by Quality Control of DNA

	**Provided saliva sample**	**Subsample assessed**	**p-values**
Subject (N)	4110	822	
Age years ± SD	14.69 ±0.30	14.69 ±0.31	0.40
Gender (%)	48.9 male	50.7 male	0.26

The mean volume of saliva collected was 2.2 mL (± 0.4), providing an average yield of 184.8 μg of DNA per subject. Larger saliva volumes collected did not directly provide greater amounts of extracted DNA (data not shown).

The first extraction yielded 100 μg of DNA (from an average of 2.0 mL Oragene DNA/saliva sample), and the second yielded 85 μg (from average 1.7 mL Oragen DNA/saliva sample). Table 2 shows the amounts of DNA obtained (ng DNA/μL) from each Oragene DNA/saliva sample. In the first extraction, each μL of Oragene DNA/saliva samples processed yielded an average of 36.6 ng DNA, whereas the second extraction yielded 47.9 ng DNA per uL (p = 1.0).

**Table 2:** DNA yield obtained with the Oragene™ DNA Self-Collection Kit in a cohort of subjects born in 1993, city of Pelotas, southern Brazil

**DNA yield (ng DNA/μL Oragene·DNA/saliva samples)**						
	**Mean ± SD**	**Median**	**Range**	**p-value *a***	**CV (%)**	**Spearman correlation (r)**
First extraction	36.6 ±23.8	30.0	2.0–160.0	1	65.2	0.84
Second extraction	47.9 ±33.6	38.5	1.0–251.0		70.1	

*a* one-tailed p-value of difference (paired *t*-test 1st >2nd extraction).

(N = 822).

The quality of DNA obtained in both extractions, evaluated through spectrophotometer readings, is shown in Table 3. Both mean and median DNA quality of the samples in both extractions remained within the recommended range of 1.6–1.8.

**Table 3:** DNA quality obtained with the Oragene™ DNA Self-Collection Kit in a cohort of subjects born in 1993, city of Pelotas, southern Brazil (N = 822)

**RAT^a^**						
	**Mean ± SD**	**Median**	**Range**	**p-value *b***	**CV (%)**	**Spearman correlation (r)**
First extraction	1.78 ± 0.12	1.79	0.84–2.64	1	6.5	0.67
Second extraction	1.84 ± 0.09	1.85	1.44–3.00		4.8	

^*a*^ RAT (Ratio of OD differences) = [(A260 – A320)/(A280 – A320)].

*b* one-tailed p-value of difference (paired *t*-test 1st >2nd evaluation).

The DNA quality evaluation by the success in amplification by PCR and RFLP-PCR reactions are summarized in Table 4. The assays displayed a rate of amplification of up to 98% in the samples.

**Table 4:** Amplification of DNA obtained by Oragene™ DNA Self-Collection Kit in a cohort of subjects born in 1993, city of Pelotas, southern Brazil (N = 822)

**Assay**	**Fragment length**	**Success rate (%)**
SNP IL4 rs2243250 by TaqMan	51 bp	99
Promoter IL4 CpG island by HRM	284 bp	99
SNP Adiponectin rs1501299C276 by PCR-RFLP	518 bp	98

## Discussion

The present study showed an average DNA yield that was 52% higher than that reported by the manufacturer [17] and higher than those described in other studies using this same kit [1,5,25,26]. However, our results were similar to results obtained by studies that used UV to measure the DNA yield [16,27]. The manufacturer admits that yields up to 200 μg can be obtained, making our average results within this range. In studies involving male subjects, older teenagers, and adults, the yields of DNA obtained were even higher than those found here [11,25]. The efficient DNA recovery here was attributable to the rubbing of the tongue inside the mouth for 15 seconds, which ensures an efficient desquamation of the oral mucosa. This would appear to agree with a study that showed that tooth brushing 30 minutes before saliva collection reduces the amount of DNA obtained [15], although our results are within the maximum range quoted by the manufacturer. Our results are that the collection of saliva close to the amount recommended by the manufacturer (4 mL) did not yield larger amounts of DNA, is corroborated by the results of Nishita et al. [11].

A comparison of DNA yield between the two extractions shows that the 8-month storage had no significant effect, because the amount of DNA obtained from each μL of Oragene·DNA/saliva samples was not lower in the second extraction, which agrees with the statement of the manufacturer [19], although it has been analyzed only the period of 8 months of storage. Thus samples can be stored for at least 8 months with no loss in the ability to obtain DNA. This was also achieved by Birnboim et al. [20], who evaluated sample viability for up to 187 days. It is noteworthy that in our study, the viability of samples was verified up to 270 days after saliva collection.

Similarly, the 8-month storage did not reduce the quality of genomic material obtained: the sample’s RAT was found to be within the recommended range of 1.6–1.8 [28] as described by the manufacturer [17]. This result agrees with those reported by a study that evaluated different storage times for the same commercial kit, but under experimental conditions [26]. The RAT was also higher than those reported for buccal swabs and FTA cards methods [5,16].

The maintenance of a sample’s viability during an 8-month storage at room temperature, together with the finding of higher RAT results in the second extraction, suggests that the kit’s reagents remains stable samples with no DNA degradation, as proposed by some works [20,29].

Successful genetic analysis for SNP genotyping depends on high DNA concentrations, but not necessarily on high yields of total DNA [25]. This is a limitation of the present study: the spectrophotometric method used in the quantitative evaluation does not allow identification of whether the DNA source is human or not. In the present study, the good amplification success of 98% was minor in PCR-RFLP assay when compared to TaqMan and HRM (99%), but was similar to results found by Koni et al. [1]. This was expected, because the technique is laborious and has many factors dependent on the DNA quality that are not measured by ultraviolet (UV) spectrophotometry. However, TaqMan and HRM assays had higher amplifications success (99%), confirming that the DNA obtained with the kit enables applications for allelic discrimination and DNA methylation. The amplifications successful found in our study suggest that the mouthwash performed 15 minutes before saliva collection reduced potential contaminants in the saliva samples, providing the expected human DNA results reported in studies with the same commercial kit [11,26,30], and showed that the DNA obtained by Oragene DNA Sample Collection Kit is suitable for genetic analysis.

## Conclusions

The present study showed that an 8-month sample saliva stored in Oragene solution at room temperature does not affect the DNA quantity and quality. It was also found that saliva collection using the Oragene kit during field work in large population-based studies allows for high DNA yields and generates DNA with adequate quality for the genetic studies required in birth cohorts and population-based studies. The collection of saliva volumes greater than 2.2 mL does not provide higher overall yield per kit. The Oragene kit is thus an effective method for obtaining high-quantity and high-quality DNA from a large number of samples.

## Competing interest

The authors state no relevant conflict of interest.

## Authors’ contributions

Ana Paula Nunes managed the saliva collection and DNA extraction, and was responsible for preparing the manuscript. Isabel Oliveira de Oliveira was in charge at the laboratory where the samples were processed. Betânia R. dos Santos, Cristini Millech, and Liziane P. Silva performed DNA extractions, readings, and data entry. David A. Gonzalez performed most of the statistical analyses. Ana M. B. Menezes, Cora L. Araújo, and Pedro C. Hallal are study coordinators of the 1993 Pelotas Birth Cohort Study in the city of Pelotas, Brazil. Fernando C. Barros is one of the main investigators of the Pelotas Birth Cohort Study in the city of Pelotas, Brazil, and assisted in the discussion section. All authors read and approved the final manuscript.

## Pre-publication history

The pre-publication history for this paper can be accessed here:

http://www.biomedcentral.com/1471-2288/12/65/prepub
